# Traditional Chinese Medicine Prescriptions Decrease Diarrhea Rate by Relieving Colonic Inflammation and Ameliorating Caecum Microbiota in Piglets

**DOI:** 10.1155/2020/3647525

**Published:** 2020-04-14

**Authors:** Jian Chen, Yaqing Mao, Chenghong Xing, Ruiming Hu, Zheng Xu, Huabin Cao, Junrong Luo

**Affiliations:** ^1^Jiangxi Provincial Key Laboratory for Animal Health, Institute of Animal Population Health, College of Animal Science and Technology, Jiangxi Agricultural University, Nanchang, Jiangxi, China; ^2^China Institute of Veterinary Drug Control (MOA Center for Veterinary Drug Evaluation), Beijing, China; ^3^Department of Mathematics and Statistics, Wright State University Dayton, Dayton, OH 45435, USA

## Abstract

Diarrhea is a leading cause of death in piglets. XiaoJianZhong (XJZ) and Jingsananli-sepsis (JSS) were two traditional Chinese medicine (TCM) prescriptions to prevent and treat intestinal diseases, including diarrhea and inflammatory disease. Here, we investigated the effects of XJZ and JSS on diarrhea rate, growth performance, colonic inflammation, and caecum microbiota in piglets. A total of 18 piglets were selected and randomly divided into three groups. Control group was supplied with basal diets, while TCM1 and TCM2 groups were, respectively, supplied with XJZ and JSS in basal diets. Decreased diarrhea rate, colonic or caecal pH, and elevated apparent nutrient digestibility were observed in both TCM groups. Meanwhile, both prescriptions alleviated colonic inflammation by decreasing mRNA expression of proinflammatory cytokines and suppressing the TLR4/MyD88/NF-*κ*B signaling pathway. Additionally, TCM1 and TCM2 prescriptions ameliorated caecum microbiota composition and increased the abundance of beneficial bacteria, together with regulations on several genes that are responsible for signaling pathways involved in cancers and metabolic diseases. Importantly, both TCM1 and TCM2 significantly promoted the average daily gain (ADG) and reduced the feed : gain (F : G) ratio. In conclusion, both TCM prescriptions effectively decreased diarrhea rate and increased growth performance by elevating apparent nutrient digestibility and gut health, via relieving colonic inflammation and ameliorating gut microbiota composition of piglets.

## 1. Introduction

It is well-known that piglets' nursery is the most critical period in the pig breeding process. However, in piglets, the gut immune system is easily disturbed because of immature immunity, deficiency of digestive ability, and enteric antigenic challenges [1]. When the external environment fluctuates, the intestinal immunity of piglets is prone to disorders leading to diarrhea, which is one of the major causes of body weight loss or even death in nursery piglets [2]. Antibiotics as feed additive have been used to promote daily gain and prevent or treat bacterial diarrhea in recent years [3]. However, the misuse and abuse of antibiotics have led to antibiotic residue and multiantibiotic resistance [4]. Thus, it is necessary to develop alternative methods to promote growth performance or antidiarrhea of piglet.

Traditional Chinese medicine (TCM) is composed of derivatives from natural plants. The pharmaceutical activities and clinical benefits of these natural ingredients have been demonstrated and witnessed for long-time clinical application [5]. Multiple active compounds in TCM could hit various targets and exert synergistic therapeutic efficacies [6]. According to the principles of TCM, typically, formulae consist of several types of medicinal herbs or minerals, in which one represents the principal component and the others serve as adjuvants to assist in the effects or facilitate the delivery of the principal component [7, 8]. Due to the complicated pathogenesis and progression of diarrhea, the therapeutic effect of a single herb may be modest in the clinic [9]. As the classical TCM prescriptions, XiaoJianZhong (XJZ) prescription and Jingsananli-sepsis (JSS) prescription are employed to prevent and treat digestive system diseases for hundreds of years, including diarrhea and enteritidis by promoting the physiological function of the spleen and stomach [8]. However, the mechanism of previously determined TCM prescriptions on ameliorating intestine health and physiology in piglets has not been clarified.

Recent study has shown that inflammatory damages toward the intestinal barrier were the main intrinsic reason of diarrhea in piglets [1]. Intestinal inflammatory response is mainly mediated by the TLR4/MyD88/NF-*κ*B signaling pathway, which enhances the production of various proinflammatory cytokines (i.e., IL-1*β*, IL-6, and TNF-*α*) [10]. On the other hand, increasing evidence highlights the cardinal role of gut microbiota in inflammatory response and immunotherapy [11, 12], due to their intrinsic capacity of drug metabolism and the influence on host metabolizing homeostasis [13]. As reported, dietary amino acids (AAs), as an ingredient in TCM, are considered as the medium for interaction between host and microorganism [14]. Meanwhile, gut microbiota have been considered as a forgotten organ for the disposition of herbal ingredients. Liu et al. [11] demonstrated a traditional Chinese herbal formula of Shen Ling Bai Zhu (some components are same as this experiment, e.g., *Poria*, *Glycyrrhiza,* and *Atractylodes*) increased the number of beneficial bacteria, such as *Bifidobacterium* and *Lactobacillus*, which may act directly or indirectly on the course of diarrhea in the gut [11]. Thus, we speculated that regulating gut microbiota composition and alleviating intestinal inflammation by TCM prescription may be a new strategy for antidiarrhea in piglets. However, the effects of all previously determined TCM prescriptions on intestinal inflammation and gut microbiota in piglets have not been clarified.

Thus, the objectives of this study were to evaluate the effects of both TCM prescriptions as feed additives on growth performance and diarrhea in piglets, and to determine whether TCM prescriptions could ameliorate health status of the large intestine by alleviating colonic inflammation response and regulating caecal microbiota.

## 2. Materials and Methods

### 2.1. Animals, Experimental Design, and Ratios

This trial was carried out in the Researching and Teaching Base of Jiangxi Agricultural University for 60 days, and the experimental design was a complete randomized design. Consistent with Shu et al. [15], diarrhea index was scored according to the fecal: 0, normal; 1, nonformed pellets; 2, soft feces; 3, semisolid containing more than half water-like feces; and 4, water-like feces. The diarrhea rate was calculated every day based on diarrhea index (diarrhea index ≥3). A total of 18 piglets (17.37 ± 1.32 kg, large white × landrace × Duroc) at 40 days of age (weaned at 5 weeks of age and 5 days for adaptation) were randomly allocated into three groups based on body weight and gender (*n* = 6). Experimental group included (a) basal diet (Control), (b) basal diet supplemented with 10 g/kg XJZ prescription (TCM1), or (c) basal diet supplemented with 3 g/kg JSS prescription (TCM2), respectively. The dose of TCM1 (10 g/kg) and TCM2 (3 g/kg) was assessed based on our preliminary tests and traditional Chinese pharmacopoeia (2005). Basal diets were formulated to meet the nutrient requirements of piglets (NRC, 2012). All the raw materials for TCM1 and TCM2 were provided by The Spirit Jinyu Biological Pharmaceutical Co., Ltd (Huhhot, Inner Mongolia, China). All dried Chinese herbs were smashed through a 2.5 mm screen sieve. Composition and main active constituents of TCM1 and TCM2 are presented in Table 1.

### 2.2. Sample Collection and Gut pH Value Detection

Fresh fecal samples (200–300 g per sample) were collected on the ground during the last 3 days of the experimental period. On the last day of the experiment, all piglets were killed by euthanasia with an intravenous injection of sodium pentobarbital (40 mg/kg·BW). Then, the abdominal cavity was opened for sample collection. Segments of midcolon tissue and midcaecum contents were harvested immediately after euthanasia, rapidly frozen in liquid nitrogen, and stored at −80°C for various analyses. Meanwhile, use a mobile pH detector (pH-STAR) to measure pH values of the midcolon and midcaecum contents. All experimental protocols were approved by the Committee for the Care and Use of Experimental Animals, Jiangxi Agricultural University, Jiangxi, China.

### 2.3. Measurement of Apparent Nutrient Digestibility

Feed and fecal samples were dried to constant weight at 65°C for 72 hours in a forced air oven. The treatment of feces and feed samples and the measurement of crude protein (CP), crude fat (CF), neutral detergent fiber (NDF), acid detergent fiber (ADF), and Ca and P apparent digestibility were consistent with the description in Guo et al. [16].

### 2.4. Determination of the Growth Performance

Daily feed intakes were recorded, and body weights of piglets on an empty stomach were measured on days 1 and 60 of the experimental period. On the basis of these data, average daily gain (ADG), average daily feed intake (ADFI), and the feed : gain (F : G) ratio were calculated.

### 2.5. Quantitative Real-Time PCR

The mRNA expression of inflammation-related genes (TLR4, MyD88, NF-*κ*B, IL-6, IL-8, IL-10, and TNF-*α*) in the TLR/MyD88/NF-*κ*B signaling pathway in the colon was determined by quantitative real-time PCR (qRT-PCR). The total RNA was isolated using the TransZol Up Reagent (TransGen Biotech, Beijing, China). In brief, cDNA was synthesized using a *TransScript*® One-Step gDNA Removal and cDNA Synthesis SuperMix reagent kit (TransGen Biotech, Beijing, China) according to the kit's instructions. Then, it was stored at −20°C for SYBR Green qRT-PCR. Gene-specific primers of all genes were designed using the Primer Premier software (PREMIER Biosoft International, CA, USA). The housekeeping gene GAPDH was used as an internal reference, and the primer sequences are shown in Table 2. The RT-qPCR profiles were as follows: 95°C for 10 minutes, 40 cycles at 95°C for 15 seconds, 60°C for 60 seconds, and extension at 95°C for 15 seconds.

### 2.6. Western Blot

Total protein was extracted with RIPA Lysis buffer (Applygen, Beijing, China). Protein supernatant was separated by 10% SDS-PAGE and transferred onto a PVDF membranes. After being blocked with 5% skimmed milk powder, the membrane was incubated with the appropriate primary antibodies overnight at 4°C, followed by incubation with the corresponding secondary antibodies for 1 hour at room temperature. The membranes were washed three times for 10 minutes each, incubated with SuperSignal chemiluminescent substrate (Pierce), and imaged by ChemiDoc XRS + Imaging System (Bio-Rad). Blots were semiquantified using BandScan software. Primary antibodies for NF-*κ*B p65 (Cell Signaling, 6956, 1 : 1000), phospho-NF-*κ*B p65 (Cell Signaling, 3033, 1 : 1000), I*κ*B-*α* (Cell Signaling, 4814, 1 : 1000), and phospho-I*κ*B-*α* (Cell Signaling, 9246, 1 : 1000) were used in this study.

### 2.7. 16S rRNA MiSeq Sequencing of Gut Microbiota

Sequencing service was provided by Personal Biotechnology Co., Ltd., Shanghai, China. Total DNA was isolated from the caecum content samples as previously reported with some modifications [17]. The V3-V4 region of bacterial 16S rRNA gene was amplified by PCR, and the products were separated by gel electrophoresis and purified using the AP-GX-500 DNA Gel Extraction Kit (Axygen, Corning, USA). Library was build up with the obtained products and then sequenced on a MiSeq sequencing platform (Illumina, USA) as described by Zhao et al. [18].

### 2.8. Bioinformatics Analysis

The trimmed and assembled sequences from each sample were aligned to the Greengene 16S rRNA database using the best hit classification option to classify the taxonomy abundance in QIIME [19]. Bacterial operation taxonomic units (OTUs) were generated using the uclust function in QIIME. A Venn diagram was generated to compare OTUs between groups. The following statistics were performed by R software. ACE, Chao, Simpson, and Shannon indices were calculated for *α*-diversity evaluation. The abundance and diversity of the OTUs (*β*-diversity) were examined using partial least squares discriminant analysis (PLS-DA) and nonmetric multidimensional scaling (NMDS) in R software. The statistical significance of the separation among groups was assessed by the linear discriminant analysis effect size (LEfSe) method based on linear discriminant analysis (LDA) scores exploited by Curtis Huttenhower (http://huttenhower.sph.harvard.edu/galaxy/), which used the nonparametric factorial Kruskal–Wallis and Wilcoxon rank sum test to identify key OTUs for separating different treatment groups at a significance level of 0.05. Microbial functions were predicted using PICRUSt, as described by Jiyan et al. [20].

### 2.9. Statistical Analysis

Statistical analysis was performed using SPSS 17.0 software (SPSS Inc., Chicago, IL, USA). All results are expressed in the format of mean ± standard deviation (SD). Comparisons between two or multiple groups were made with Student's *t*-test or ANOVA. *P* < 0.05 was considered to be significant, and *P* < 0.01 was considered to be highly significant.

## 3. Results

### 3.1. Diarrhea Index

**Figure 1:**
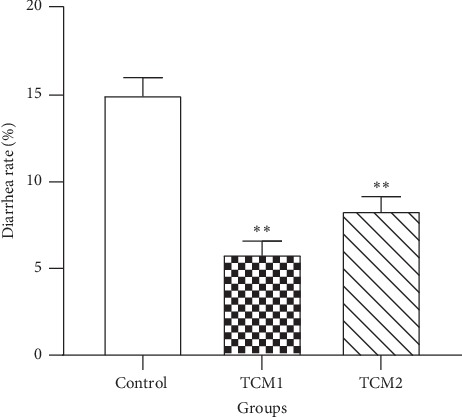
Effects of TCM supplements on diarrhea rate of piglets. *∗∗P* < 0.01 vs. the control. The results are expressed as mean ± SD, one-way ANOVA, *n* = 6.

Diarrhea is the main cause of the decline in the survival rate of nursery piglets. To determine whether TCM could effectively prevent piglets' diarrhea, we calculated the diarrhea rate (Figure 1). After 60 days of treatment, both TCM groups significantly reduced the diarrhea rate in piglets. TCM1 and TCM2 showed a significant reduction of the diarrhea rate compared to the control (*P* < 0.01). However, there was no significant difference between TCM1 and TCM2 treatment (*P* > 0.05).

### 3.2. Intestinal pH Value

More acidic intestinal environment could inhibit the proliferation of various pathogens. To determine whether TCM could ameliorate the environment of large intestine, we measured the colon and caecum pH value. Compared to the control, colon and caecum pH were decreased in different levels after TCM intervention (Table 3). Moreover, TCM1 reduced the colon and caecum pH by 7.85% (*P* < 0.05) and 11.54% (*P* < 0.01), but no difference was noticed on the colon pH of the TCM2 group (*P* > 0.05) compared to the control. Similarly, the caecum pH also declined in the TCM2 group extremely significantly (*P* < 0.01).

### 3.3. Nutrient Apparent Digestibility

To evaluate whether both TCM treatments could improve the apparent digestibility in piglets, we assessed the digestibility of CP, CF, NDF, ADF, Ca, and P (Table 4). Compared to the control, TCM1 notably improved the apparent nutrient digestibility of CP, CF, NDF, ADF, Ca, and P by 5.04%, 6.63%, 12.04%, 17.12%, 6.56% (*P* < 0.01), and 6.31% (*P* < 0.05), respectively. Similarly, the results of CP, NDF, and ADF were elevated by 2.56%, 7.10% (*P* < 0.05), and 13.64% (*P* < 0.01) in the TCM2 group, but no significant difference was observed in CF, ADF, Ca, and P levels (*P* > 0.05).

### 3.4. Growth Performance

To evaluate the effects of TCM1 and TCM2 on growth performance in piglets, we measured the ADG, ADFI, and F : G ratio (Table 5). Compared to the control, ADG was significantly increased in both treatment groups (*P* < 0.05), whereas no difference of ADFI was observed among all groups. Meanwhile, both TCM1 and TCM2 significant decreased the F : G ratios (*P* < 0.01).

### 3.5. mRNA Expression of TLR4/MyD88/NF-*κ*B Signaling Pathway

**Figure 2:**
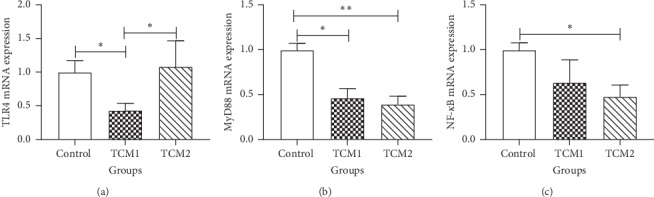
Effects of both TCM supplements on mRNA expression of (a) TLR4, (b) MyD88, and (c) NF-*κ*B in the colon of piglets. “*∗*” and “*∗∗*” indicate a significant difference (*P* < 0.05) or a highly significant difference (*P* < 0.01) between treatment groups, respectively. The results are expressed as mean ± SD, one-way ANOVA, *n* = 6.

To determine whether the colonic inflammatory response was alleviated after the TCM intervention, we analysed the mRNA levels of inflammation-related genes in the TLR4/MyD88/NF-*κ*B signaling pathway. As shown in Figure 2, compared to the control, the TLR4 mRNA level was only downregulated significantly in the TCM1 group (*P* < 0.05), but there was no significant difference in the TCM2 group (*P* > 0.05). Notably, the mRNA expression of MyD88 was decreased significantly in the TCM1 group (*P* < 0.05) and TCM2 group (*P* < 0.01). Additionally, NF-*κ*B levels of both TCM groups were decreased, but only the TCM2 group showed statistical significance to the control group (*P* < 0.05).

### 3.6. mRNA Expression of Inflammatory Cytokine Genes

**Figure 3:**
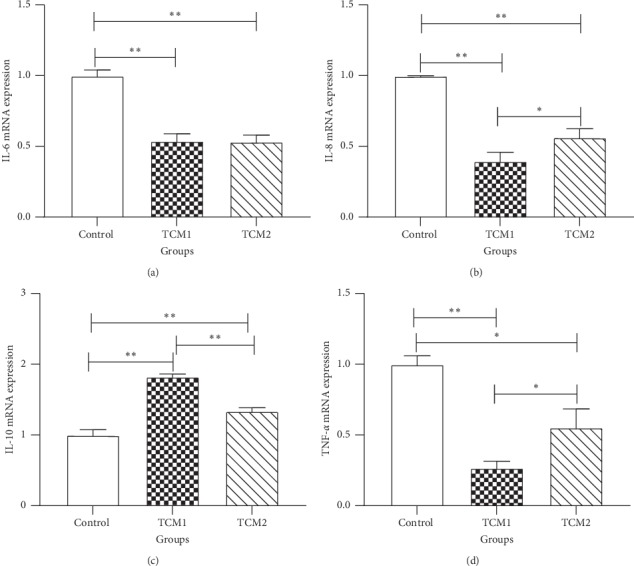
Effects of both TCM supplements on mRNA expression of inflammatory cytokines in the colon of piglets: (a) IL-6; (b) IL-8; (c) IL-10; (d) TNF-*α*. “*∗*” and “*∗∗*” indicate a significant difference (*P* < 0.05) or a highly significant difference (*P* < 0.01) between treatment groups, respectively. The results are expressed as mean ± SD, one-way ANOVA, *n* = 6.

To evaluate the levels of inflammatory cytokines downstream of the TLR4/MyD88/NF-*κ*B signaling pathway, the mRNA expression of IL-6, IL-8, IL-10, and TNF-*α* was measured (Figure 3). Compared to the control, the expression of IL-6, IL-8, and TNF-*α* was decreased, whereas IL-10 levels were increased significantly in both TCM prescriptions (*P* < 0.05 or *P* < 0.01). Moreover, IL-8 and TNF-*α* levels were decreased, and IL-10 levels increased significantly (*P* < 0.05) in the TCM1 group, compared to the TCM2 group.

### 3.7. Protein Levels of NF-*κ*B Signaling Pathway in the Colon

**Figure 4:**
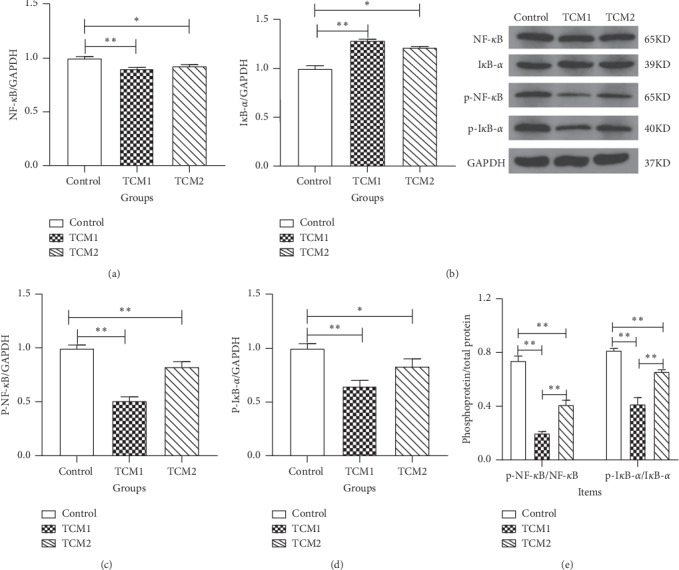
Effect of both TCM supplements on protein expression of NF-*κ*B signaling pathway in colon of piglets: (a) NF-*κ*B/GAPDH; (b) I*κ*B-*α*/GAPDH; (c) p-NF-*κ*B/GAPDH; (d) p-I*κ*B-*α*/GAPDH; (e) phosphoprotein/total protein. “*∗*” and “*∗∗*” indicate a significant difference (*P* < 0.05) or a highly significant difference (*P* < 0.01) between treatment groups, respectively; the results are expressed as mean ± SD, one-way ANOVA, *n* = 6.

To further determine the inhibition of NF-*κ*B signaling pathway in the colon after treating with TCM supplements, the protein levels of NF-*κ*B p65, p-NF-*κ*B p65, I*κ*B-*α*, and p-I*κ*B-*α* were analysed. Compared to the control, both TCM1 and TCM2 that were highly significant decreased the protein levels of NF-*κ*B p65, p-NF-*κ*B p65, and p-I*κ*B-*α* (*P* < 0.05 or *P* < 0.01), respectively (Figure 4), while that of I*κ*B-*α* protein level was remarkably elevated in the TCM1 (*P* < 0.01) and TCM2 (*P* < 0.05) groups. Importantly, both TCM1 and TCM2 reduced the ratio of phosphorylated protein (p-NF-*κ*B and p-I*κ*B-*α*) to total protein significantly (*P* < 0.01). Meanwhile, the ratio of p-NF-*κ*B/NF-*κ*B and p-I*κ*B-*α*/I*κ*B-*α* of the TCM2 group was remarkably higher than the TCM1 group.

### 3.8. Caecum Microbiota DNA Sequence Data

**Figure 5:**
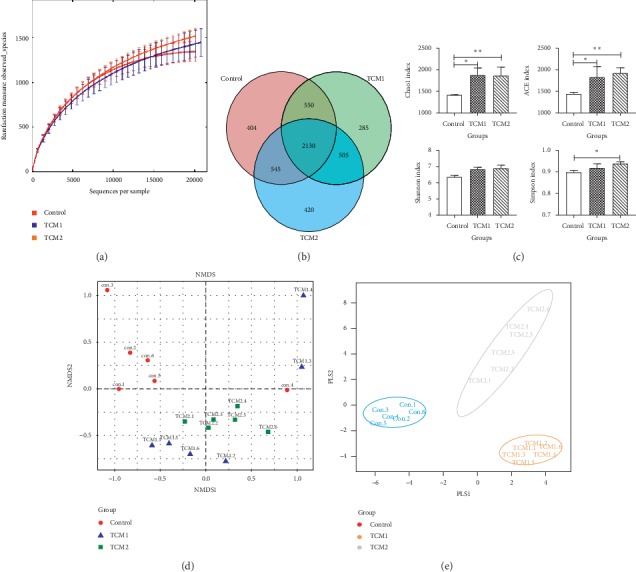
*α*-Diversity and *β*-diversity analysis on the caecum microbiota. (a) Rarefaction curves; red, blue, and orange indicate the 6 samples of control, TCM1, and TCM2 groups, respectively. (b) Venn diagram; each circle represents a set of samples; the group between the circle and circle overlapping part digital represents of the common OTUs, and there is no overlapping part representing unique OTUs in each group. (c) Alpha diversity indices comparison; the ACE and Chao1 indexes represent the community richness of the microbiota, and the Shannon and Simpson indexes represent the community diversity of the microbiota. Results are expressed as mean ± SD, one-way ANOVA, *n* = 6. ^*∗*^*P* < 0.05 vs. the control; ^*∗∗*^*P* < 0.01 vs. the control. (d) UniFrac distance-based nonmetric multidimensional scaling (NMDS); red circle, the control group; blue triangle, the TCM1 group; green square, the TCM2 group. The points of different colors belong to different samples (groups). Each point represents one sample. The closer the distance between two points, the higher the similarity, and the smaller the difference in the microbial community structure between the two samples. (e) Partial least squares discriminant analysis (PLS-DA); each point represents a sample, points of the same color belong to the same group, and points of the same group are marked with ellipses. If the samples belonging to the same grouping are closer to each other and the distance between the points of different grouping is farther, the classification model is better.

To evaluate the effect of TCM1 and TCM2 prescriptions on the caecum microflora, we performed 16s RNA sequencing on the caecum contents of piglets. After quality control on the original sequence, 763938 valid sequences in total (on average 42441 sequences per sample) were obtained. Rarefaction analysis results showed that this sequencing depth was sufficient to study the microbial diversity of each sample (Figure 5(a)). Common OTU analysis presented by Venn diagram indicated that there were 404 unique OTUs in the control, 285 in the TCM1 group, and 420 in the TCM2 group, respectively, while 2130 common OTUs were identified in all samples (Figure 5(b)).

### 3.9. *α*-Diversity and *β*-Diversity of the Caecum Microbiota

As shown in Figure 5(c), the Shannon and Simpson indexes of TCM1 showed an higher species diversity than control, but the difference was not significant (*P* > 0.05). The ACE and Chao1 indexes of both TCM1 and TCM2 were significantly higher than the control group (*P* < 0.05). Meanwhile, TCM2 elevated (*P* < 0.05) the Simpson index significantly compared to the control.

*β*-Analysis was used to compare the similarity of overall community structure, which employed several unsupervised multivariate statistical assessments, including UniFrac NMDS and PLS-DA. The *β*-diversity maps showed that the similarity in species diversity was remarkably different between the both TCM groups and the control group. As shown in Figures 5(d) and 5(e), there was an apparent distance of piglet caecum flora among the TCM1, TCM2, and control group. In addition, the group spacing in the TCM treatment groups was smaller than that between the treatment groups and the control group.

### 3.10. Key Community of Caecum Microbiota

**Figure 6:**
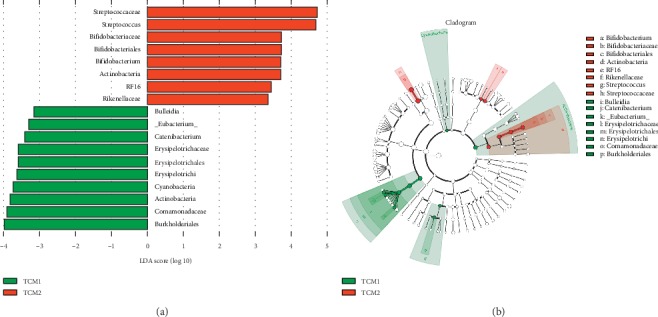
Linear discriminant analysis effect size (LEFse) analysis on caecum microbiota. (a) Linear discriminant analysis (LDA): an LDA score higher than 2 indicated a higher relative abundance in the corresponding group than in the other two groups. (b) LEfSe taxonomy cladogram: different colors suggest enrichment of certain taxa in TCM1 (blue) and TCM2 (red). The size of the circles is based on relative abundance. The significantly different species using the nonparametric factorial Kruskal–Wallis rank sum test at a significance level of 0.05.

LDA score (Figure 6(a)) higher than 2 indicated a higher relative abundance in the corresponding group than in the other two groups (*P* < 0.05). The relative abundance of significantly different species (*P* < 0.05) was showed by LEfSe taxonomy cladogram (Figure 6(b)). The significantly different communities (*P* < 0.05) belonged to *Cyanobacteria* and *Actinobacteria* when supplementation of TCM1. At the genus level, TCM1 showed significant (*P* < 0.05) selective enrichment of *Eubacterium*, *Bulleidia,* and *Catenibacterium*, with the LAD scores of 3.31, 3.16, and 2.88, respectively, and TCM2 demonstrated a significant (*P* < 0.05) effect on *Streptococcus* and *Bifidobacterium*, with the LAD scores of 4.69 and 3.71, respectively.

### 3.11. Microbiome Function Regulation by TCM Treatment

**Figure 7:**
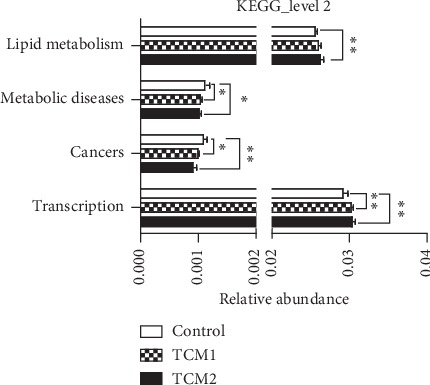
Microbiome function prediction according to the KEGG pathway database. The horizontal coordinates represent the relative abundance of functional genes in different groups; the vertical coordinates represent the functions of genes. The “*∗*” or “*∗∗*” indicates a significant difference (*P* < 0.05) or a highly significant difference (*P* < 0.01) between treatment groups, respectively.

Comparing the sequencing data with KEGG pathway database by PICRUSt (Figure 7), it was found that both TCM groups significant upregulated (*P* < 0.05) the abundances of genes responsible for transcription (Genetic Information Processing), but downregulated (*P* < 0.05 or *P* < 0.01) genes involved in the “cancers” and “metabolic diseases” pathways. Meanwhile, TCM2 also significantly upregulated (*P* < 0.01) abundant genes responsible for lipid metabolism.

## 4. Discussion

The results in this study demonstrated that dietary supplementation with both TCM prescriptions could reduce diarrhea rate, promote growth performance, suppress TLR4/MyD88/NF-*κ*B signaling pathways, and decrease the levels of proinflammatory cytokine expression in the colon of piglets. Accordingly, the 16S rRNA analysis revealed that the component and relative abundances of beneficial bacteria were altered in both TCM groups, compared to the control.

During the nursing period, piglets are prone to colonic dysfunction, leading to diarrhea and edema, which results in impaired growth performance or even death [21, 22]. Previous study reported that supplementation of medicinal plants or its extracts increases growth performance in pigs, mainly because the active ingredient could have antimicrobial properties, aid in the decrease in intestinal pH and against diarrhea, and improve nutrient digestibility [22, 23]. Meanwhile, reducing the pH value of gut contents could inhibit or eliminate some pathogenic flora to prevent diarrhea via improving gut health status in piglets. In this study, both TCM prescriptions significantly decreased the diarrhea rate and colon or caecum pH value in piglets, which suggested that TCM prescriptions were effective for diarrhea control, and it may attribute to the TCM capable of improving the intestinal environment directly or indirectly.

Weaning or other stresses can damage the growth performance of piglets, via decreasing the ADG and ADFI. In this study, the difference was not significant in the ADFI among all groups, which may be because the TCM additives slightly changed the taste of the feed. Notably, our results indicated that both TCM1 and TCM2 promoted ADG and decreased the F : G ratio remarkably, indicating that both treatments promoted growth performance in piglets. The improvement of growth performance is associated with better intestinal environment (intestinal pH) and the promotion of energy intake by elevating apparent nutrient digestibility. Meanwhile, intestinal peristalsis improvement and acidic environment contribute to increasing the digestion and absorption of nutrients and improving the nutritional digestibility of piglets, subsequently promoting growth [24], which is of great significance for piglets with the imperfect digestive system. Huang et al. suggested that TCM supplementation increased the apparent nutrient digestibility such as dietary dry matter, CP, and gross energy in pigs, which is consistent with our results [24]. Moreover, our data showed that the digestibility of CP, CF, NDF, ADF, Ca, and P was increased in TCM1 treatment, but supplementation with TCM2 has no significant effect on CF, Ca, and P absorption, indicating that TCM1 exerts a more comprehensive effect on apparent digestibility than that of TCM2. Additionally, the changes in various nutrient digestibility may be also associated with the alleviation in intestinal inflammation and improvement of gut microbiota, much likely due to TCM1 and TCM2.

It is well established that different regions of the gut have distinct physiological functions. Large intestine has optimal conditions for prolific bacterial growth: warm, moist, anaerobic, and filled with feed residues that flow at a relatively low speed. Many kinds of pathogens, such as spirochetes, pass through the small intestine until ultimately colonizing and proliferating in the colon, which increases the risk of bacterial infection and inflammatory response [25, 26]. Meanwhile, previous study has demonstrated that TLR4 and CD14 are expressed higher in the colon than intestine [27]. Thus, given the highest correlation between colon immunity with TLR4/MyD88/NF-*κ*B signaling pathway, we examined the expression of inflammation-related genes and proteins in the TLR4/MyD88/NF-*κ*B signaling pathway, as well as the inflammatory cytokines downstream.

Although there are various causes of diarrhea, intestinal inflammation is usually involved. The TLR4/MyD88/NF-*κ*B signaling pathway is one of the major pathways mediating inflammatory responses in the intestine, which is triggered by pattern recognition receptors (PRRs) and TLRs via interacting with the antigen from gut microbiota [28]. TLR4 is essential for LPS-mediated signaling. After binding to TLR4, LPS activates the MyD88-dependent pathway, a key inflammatory signaling pathway [29]. The activation of MyD88 will phosphorylate I*κ*B kinase, which in turn activates the transcription factor NF-*κ*B and triggers the downstream expression of proinflammatory cytokines along with other immune-related genes [30]. NF-*κ*B resides outside the nucleus in its inactive form bound to inhibitory I*κ*B-*α*. Upon activation, the protein levels of NF-*κ*B p65, p-NF-*κ*B p65, and p-I*κ*B-*α* were increased and the levels of I*κ*B-*α* were decreased [31]. In this study, both TCM treatments downregulated NF-*κ*B p65, p-NF-*κ*B p65, and p-I*κ*B-*α* protein levels, upregulated the I*κ*B-*α* protein levels, and significantly reduced the ratio of phosphorylated protein (p-NF-*κ*B p65 and p-I*κ*B-*α*) to total protein, which is consistent with Xu et al. [32], thus endorsing the anti-inflammatory activity of TCM1 and TCM2. Importantly, in TCM groups, the results of inflammation-related genes in the TLR4/MyD88/NF-*κ*B signaling pathway and NF-*κ*B p65, p-NF-*κ*B p65, I*κ*B-*α*, and p-I*κ*B-*α* protein levels were in agreement with the lower levels of inflammatory cytokine, which indicated that TCM relieved inflammation by suppressing the activity of TLR4/MyD88/NF-*κ*B signaling pathway in the colon. Moreover, these changes may be associated with the TCM-induced amelioration of gut microbiota diversity or abundance [33].

The interaction between microbiota and the host influences immunological homeostasis, and the changes in this interaction are associated with various inflammatory cytokines. Previous study has proved that TCM prescription decreased inflammatory cytokine level that was related to the increased abundance of beneficial gut microbiota and decreased LPS levels [34]. Furthermore, certain components in Chinese herbal medicines exert their pharmacological effects through interactions with gut bacteria [14, 35]. Importantly, disturbed gut microbiota, in either diversity or abundance, have been found to play an important role in the pathological development of inflammatory bowel disease (IBD) [33]. In present study, TCM groups significantly elevated the richness or diversity of caecum microbiota. Also, according to the NMDS and PLS-DA analysis, we observed significant structural changes in the caecum microbiota in TCM1 and TCM2 groups compared to the control. All of these results indicated that experimental groups TCM1 and TCM2 exhibited different caecum microbiota structures compared to the control group.

**Figure 8:**
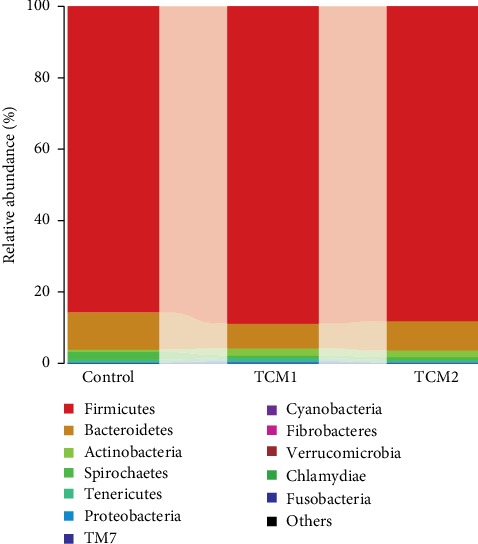
Bacterial composition of the different groups at the phylum level.

Our results showed that the main phyla were *Firmicutes*, followed by *Bacteroidetes* in caecum bacterial communities of piglets (Figure 8), which is in accordance with previous study [35]. The study indicated that supplementation of TCM or its extracts can ameliorate the structure of gut microbiota by increasing probiotics and reducing pathogens, leading to decreased risk of IBD [36, 37]. In current study, TCM1 significantly increased the caecum phylum *Cyanobacteria* and *Actinobacteria*. As reported, *Cyanobacteria* play a vital role in inhibiting inflammation by production of anti-inflammatory pitinoic acids B and C [38]. Meanwhile, at the genus level, TCM1 increased the abundance of *Eubacterium*, *Bulleidia*, and *Catenibacterium*, respectively, and that of *Streptococcus* and *Bifidobacterium* were elevated in the TCM2 group. Gut probiotics may be effective for secondary prevention in patients with recurrent *Clostridium difficile*-associated diarrhea through maintenance of the normal gastrointestinal flora [39]. As reported, *Bulleidia* could ferment glucose and anti-inflammatory products acetate, lactate, and trace amounts of succinate were the end products of fermentation [40]. A few studies demonstrated that *Eubacterium* and *Bifidobacteria* were able to use carbohydrate for the production of short-chain fatty acids, which can inhibit inflammatory responses in the gut [41, 42]. *Bifidobacteria* are also demonstrated to modulate the intestinal immune system and have been shown to increase the production of immunoglobulin A [42]. Apparently, an increase in the abundance of these beneficial bacteria species may be related to the alleviation in intestinal inflammation by TCM treatments. Conversely, intestinal bacteria can also alleviate inflammation by inducing improvements to intestinal epithelial barrier integrity [43]. Furthermore, this also may be associated with the AAs, a rich component of TCM, such as aspartic acid and glutamic acid, could be available to the microbiota in the gut in mammals, and play a critical role in regulating the intestinal mucosal immunity and microbiota directly or indirectly, contributing to intestinal homeostasis [14, 44].

Indeed, gut microbiota not only experiences long-term coevolution with host [45] but also regulates the host immunity [46]. Especially, lifestyle, diets, and medicine intake contributes greatly to the alteration of gut microbiota [47]. In current research, it was found that TCM1 and TCM2 are mainly composed of antibacterial and antitumor active ingredients such as volatile oil, alkaloids, and flavone. Thus, we explored microbiota function based on inferred metagenomes using the PICRUSt algorithm [48]. Our analysis of inferred metagenomes revealed decreased abundance of human disease pathways, including the metabolic diseases and cancers in TCM treatments compared to the control. This finding suggests that, aside from decreasing intestinal pH and inhibiting inflammation, increasing abundance of beneficial bacteria species after TCM intervention is also known to have prominent effects on metabolic diseases and cancers.

## 5. Conclusion

Dietary supplementation with TCM1 and TCM2 effectively reduced the diarrhea rate in piglets and promoted growth performance by improving the gut physical circumstance, nutrient digestibility, colonic inflammation alleviation, and caecum microbial composition amelioration. Importantly, TCM prescription may be a potential therapeutic strategy for piglet diarrhea.

## Figures and Tables

**Table 1:** Composition and main active constituents of TCM1 and TCM2 (air-dried basis)^†^.

Latin name	Main active constituent	Used part	Content (%)
TCM1			
*Cassia twig*	Cinnamaldehyde	Dried twig	13
*Glycyrrhiza uralensis*	Glycyrrhizin	Dried root	4
*Ziziphus zizyphus*	*Jujuba* polysaccharide	Dried fructification	4
*Cynanchum otophyllum*	Paeoniflorin	Dried root	13
*Zingiber officinale Roscoe*	Ginger oleoresin	Dried root	6
*Rhizoma Atractylodes*	Atractylodine	Dried root	14
*Atractylodes macrocephala*	Biatractylolide	Dried root	10.5
*Poria cocos*	Pachymaran	Dried sclerotium	10.5
*Coptis chinensis Franch.*	Berberine	Dried root	4
Maltose	Maltose	—	21
Total			100

TCM2			
*Nepeta cataria L.*	*Nepeta cataria* oil	Dried stem	16.5
*Radix Saposhnikoviae*	Chromone glycoside	Dried root	16.5
*Notopterygium incisum*	Notopterol	Dried root	16.5
*Radix Angelicae pubescentis*	Heraclenin	Dried root	16.5
*Radix bupleuri*	Saikosaponin	Dried root	10
*Radix Peucedani*	Peucedanin	Dried root	10
*Poria cocos*	Pachymaran	Dried sclerotium	10
*Glycyrrhiza uralensis*	Glycyrrhizin	Dried root	4
Total			100

*Note.* ^†^Main active constituents of TCM come from Chinese pharmacopoeia (2005).

**Table 2:** Primers used in this study.

Target	GenBank number	Primers sequence (5′-3′)
GAPDH	NM_001206359	F: ACTCACTCTTCCACTTTTGATGCT
		R: TGTTGCTGTAGCCAAATTCA
TLR4	NM_001113039.1	F: GCCATCGCTGCTAACATCATC
		R: CTCATACTCAAAGATACACCATCGG
MyD88	NM_001099923.1	F: TGGTAGTGGTTGTCTCTGATGA
		R: TGGAGAGAGGCTGAGTGCAA
NF-*κ*B	NM_001048232.1	F: CTCGCACAAGGAGACATGAA
		R: ACTCAGCCGGAAGGCATTAT
IL-6	NM_001252429.1	F: TGGCTACTGCCTTCCCTACC
		R: CAGAGATTTTGCCGAGGATG
IL-8	NM_213867.1	F: TTCGATGCCAGTGCATAAATA
		R: CTGTACAACCTTCTGCACCCA
IL-10	HQ026020.1	F: GCTGAAGACCCTCAGGCTGA
		R: GCTGAAGACCCTCAGGCTGA
TNF-*α*	NM_214022.1	F: CCAATGGGCAGAHTGGGTATG
		R: TGAAGAGGACCTGGGAGTAG

*Note.* GAPDH, glyceraldehyde-3-phosphate dehydrogenase; TLR4, toll-like receptors 4; MyD88, myeloid differentiation factor 88; NF-*κ*B, nuclear factor-*κ*B; IL-6, interleukin-6; IL-8, interleukin-8; IL-10, interleukin-10; TNF-*α*, tumor necrosis factor-*α*.

**Table 3:** Effects of TCM supplements on intestinal pH in piglets.

Items	Groups		
	Control	TCM1	TCM2
Colon pH	6.62 ± 0.09^a^	6.10 ± 0.14^b^	6.50 ± 0.21^ab^
Caecum pH	6.76 ± 0.07^A^	5.98 ± 0.10^B^	6.17 ± 0.06^B^

*Note*. Results are expressed as mean ± SD, one-way ANOVA, *n* = 6. ^a,b^Within a row, means without a common lowercase superscript are different at *P* < 0.05. ^A,B^Within a row, means without a common uppercase superscript are different at *P* < 0.01.

**Table 4:** Effects of TCM supplements on nutrient digestibility in piglets.

Items	Groups		
	Control	TCM1	TCM2
CP (%)	74.55 ± 0.34^Aa^	78.51 ± 0.61^Bc^	76.51 ± 0.30^Ab^
CF (%)	73.50 ± 0.67^A^	78.72 ± 0.58^B^	74.57 ± 0.66^A^
NDF (%)	53.39 ± 1.03^Aa^	60.70 ± 1.22^Bb^	57.47 ± 0.99^ABb^
ADF (%)	25.90 ± 0.67^A^	31.25 ± 0.62^B^	29.99 ± 0.97^B^
Ca (%)	58.42 ± 0.73^A^	62.52 ± 0.73^B^	60.50 ± 0.89^AB^
P (%)	48.83 ± 1.00^a^	52.12 ± 0.75^b^	49.26 ± 1.01^a^

*Note*. CP, crude protein; CF, crude fiber; NDF, neutral detergent fiber; ADF, acid detergent fiber. Results are expressed as mean ± SD, one-way ANOVA, *n* = 6. ^a,b^Within a row, means without a common lowercase superscript are different at *P* < 0.05. ^A,B^Within a row, means without a common uppercase superscript are different at *P* < 0.01.

**Table 5:** Effects of TCM treatments on growth performance in piglets.

Items	Groups		
	Control	TCM1	TCM2
ADG (kg)	0.52 ± 0.01^a^	0.56 ± 0.03^b^	0.56 ± 0.02^b^
ADFI (kg)	0.95 ± 0.03	0.98 ± 0.07	1.00 ± 0.05
F : G (kg/kg)	1.85 ± 0.06^A^	1.75 ± 0.05^B^	1.79 ± 0.03^AB^

*Note*. ADG, average daily gain; ADFI, average daily feed intake; F : G: feed: gain. Results are expressed as mean ± SD, one-way ANOVA, *n* = 6. ^a,b^Within a row, means without a common lowercase superscript are different at *P* < 0.05. ^A,B^Within a row, means without a common uppercase superscript are different at *P* < 0.01.

## Data Availability

The data used to support the findings of this study are available from the corresponding author upon request.
